# *Streptococcus dysgalactiae* subsp. *equisimilis* bacteremia in Finland: changes in *emm-*type distribution combined with clinical presentation

**DOI:** 10.1186/s12879-025-11875-6

**Published:** 2025-11-14

**Authors:** Viivi Nevanlinna, Janne Aittoniemi, Reetta Huttunen, Hanne-Leena Hyyryläinen, Tapio Seiskari, Tiina Luukkaala, Sari Rantala

**Affiliations:** 1 Department of Internal Medicine, Tampere University Hospital, Tampere, Finland; 2 Faculty of Medicine and Health Technology, Tampere University, Tampere, Finland; 3 Fimlab Laboratories, Tampere, Finland; 4 Public Health, Finnish Institute for Health and Welfare, Microbiology, Helsinki, Finland; 5 Research, Development and Innovation Center, Tampere University Hospital, Tampere, Finland; 6 Health Sciences, Faculty of Social Sciences, Tampere University, Tampere, Finland

**Keywords:** Streptococcus dysgalactiae subspecies equisimilis; SDSE; Bacteremia; Emmtype

## Abstract

**Background:**

The incidence of SDSE bacteremia has risen dramatically over the past two decades, but it is unclear whether this increase is influenced by changes in the *emm* types of SDSE isolates. Furthermore, the relationships between *emm* types and clinical manifestations or disease severity are poorly understood.

**Methods:**

We conducted a retrospective, population-based study of *emm* types in SDSE bacteremia episodes in the Pirkanmaa health district from August 2015 to June 2018 and compared the findings to data from the same geographic area collected between 1995 and 2004.

**Results:**

During the study period, 230 episodes of SDSE bacteremia were identified. The most common *emm* types were stG480, stG485, and stG6, consistent with findings from the same geographic area 20 years ago. The most notable change in *emm* type distribution was the emergence of stG62647 as the third most common *emm* type, along with stG6. Despite changes in distribution, the incidence rates of isolates of all *emm* types identified in this study have significantly increased compared with the study conducted 1995 to 2004. *Emm* type StC74a was associated with higher mortality rates (OR 4.088 [95% CI 1.177–14.202]). Skin and soft-tissue infections were less common with stG485 (OR 0.455 [95% CI 0.207–1.000]), and the infection focus was more often unknown (OR 3.395 [95% CI 1.375–8.382]). StG480 was associated with arthritis (OR 5.147 [95% CI 1.227–21.589]).

**Conclusions:**

The increase in SDSE bacteremia is driven by both the rise of formerly common *emm* types and the newly emerged strain stG62647.

## Introduction

*Streptococcus dysgalactiae* subspecies *equisimilis*(SDSE) is a beta-hemolytic streptococcus that primarily expresses Lancefield carbohydrate antigens C and G [1]. SDSE is part of the human commensal flora and was once considered to be of low pathogenicity [2]. However, more recent studies have shown that it causes a range of infections similar to those of *Streptococcus pyogenes*, including severe invasive infections such as cellulitis, pneumonia, deep abscesses, arthritis, endocarditis, and necrotizing fasciitis [3]. 

The incidence of SDSE bacteremia has significantly increased over the past few decades, even surpassing that of *S. pyogenes* in some countries [4–7]. This rise has been particularly notable in Finland, where the incidence of Group C and G bacteremia reached 17.6/100,000 inhabitants in 2019 [8]. A similar trend was observed in the Pirkanmaa Health District (HD), with the incidence of SDSE bacteremia rising from 2.05/100,000 in 1995 to 16.9/100,000 inhabitants in the current study population in 2015–2018 [9, 10]. 

In a genome sequence homology analysis, SDSE has been closely related to *S. pyogenes*, sharing 72% genetic similarity, presumably due to a common ancestor, but also horizontal gene transfer [11, 12]. *S. pyogenes* and SDSE share many major virulence factors, including the M-protein, which is essential for resisting phagocytosis, disrupting the complement system and promoting adherence to human cells [13]. As in *S. pyogenes*, the N-terminal region of the *emm* gene, encoding the M-protein, is also used for molecular typing in SDSE.

In Finland, the most common *emm* types in 1994–2004 were stG480, stG6, and stG485, which have also been among the most common isolates in Norway and Sweden [14–16]. However, in the past two decades, new regional upsurges in certain *emm* types have been reported. In several European countries, including Norway, Denmark, Spain, and Germany, as well as in Canada, the lineage stG62647 has become the major *emm* type, whereas in Japan, stG6792 became predominant in the 2000 s [5, 17–21]. The *emm* type stG62647 has been associated with severe clinical manifestations and higher case fatality rates in Norway [17]. Similarly, stG6792 has been associated with poor outcomes in Japan [22]. While specific *emm* types in *S. pyogenes* have been associated with certain clinical manifestations, similar studies on SDSE bacteremia are still lacking. In one previous study of SDSE bacteremia, no association was found between *emm* type and the source of infection [23]. 

In this study, we provide an updated distribution of *emm* types in SDSE bacteremia, combined with clinical data, in the Pirkanmaa Health District, Finland. Our objective was to compare these findings with previously reported data from the same health district covering the years 1995–2004 [9, 14]. We aimed to investigate whether the changes in *emm* type-distribution contribute to the substantial increase in the incidence of SDSE bacteremia. Moreover, we present, to our knowledge, the first comprehensive study evaluating the associations between *emm* types and disease severity, as well as *emm* types and clinical manifestations.

## Methods

The Pirkanmaa Health District is the second-largest health district in Finland, with a catchment area of approximately 535 000 inhabitants. It includes Tampere University Hospital, four regional hospitals, and various smaller healthcare units. This population-based study was focused on all adult patients (≥ 18 years old) with blood culture positive SDSE bacteremia within Pirkanmaa HD from August 2015 to July 2018. All positive blood cultures were recorded in the Finnish Register for Hospital Infections and Antimicrobial Use (SAI) and were identified retrospectively. Throughout the study period, 230 episodes involving ≥ 1 positive blood cultures for SDSE were identified, with all patients also exhibiting clinical signs compatible with infection. Of these episodes, *emm* typing was conducted on 229 SDSE isolates, and medical records were available for 217 episodes (211 patients). The medical records were reviewed, and structured case report forms (SRs) were completed. The study was approved by the Regional Ethics Committee of Tampere University Hospital.

All blood samples taken at Pirkanmaa HD were studied at Fimlab laboratories, Tampere. During August 2015–October 2017, blood-culture samples were collected into BacT/Alert FA Plus aerobic and FN Plus anaerobic blood-culture bottles and incubated in an automated BacT/Alert 3D microbial detection system (bioMérieux). During November 2017– July 2018, blood-culture samples were collected in BD BACTEC Plus Aerobic/F and Lytic/10 Anaerobic/F culture vials and incubated in a BD BACTEC FX blood-culture system (Becton Dickinson).

SDSE was primarily identified on the basis of typical large-colony-forming growth and β-hemolysis on blood agar plates. Until February 2017, identification of the bacteria was based on latex agglutination (Lancefield grouping, PathoDxtraTM Strep Grouping Kit, Thermo Scientific, Basingstoke, Hants, UK), with confirmation (API^®^ 20 STREP [bioMérieux, Marcy l’Etoile, France] or matrix-assisted laser-desorption/ionization time-of-flight mass spectrometry, i.e., MALDI-TOF MS [VITEK^®^ MS, bioMérieux, Marcy l’Etoile, France]). Since March 2017, MALDI-TOF MS has been the primary method for identification. MALDI-TOF analysis provides results for *S. dysgalactiae* subsp. *dysgalactiae/equisimilis*, which was interpreted as *S. dysgalactiae* subsp. *equisimilis* associated with human disease. Antimicrobial susceptibility testing was based on the disk diffusion method, according to The European Committee on Antimicrobial Susceptibility Testing (EUCAST) standard [24]. The SDSE strains were stored in skimmed-milk broth at −70 °C until further *emm* type analysis. *Emm* typing was conducted according to the guidelines of the Centers for Disease Control and Prevention [25]. 

Epidemiology, patient characteristics, and clinical manifestations of the study population have been described in detail elsewhere [10, 26]. To compare the distribution of *emm* types, we utilized previously published data from the same health district, covering the years 1995–2004 [9, 14]. Statistical analyses were performed by using IBM SPSS Statistics for Mac, Version 29 (IBM corp., Armonk, NY, US). The associations between *emm* types and sex, clinical manifestations and markers of severe disease were tested using the χ2 test or Fisher’s exact test, as appropriate. The associations between emm types and age was studied using the Mann–Whitney U test. P-values under 0.05 were considered statistically significant. Mantel–Haenszel Common Odds Ratio estimates were calculated, and odds ratios are expressed with 95% confidence intervals.

## Results

Over the three-year study period, 230 episodes of SDSE bacteremia were detected, with 229 episodes available for *emm* typing. Medical records were available concerning 217 episodes, involving 211 patients. Altogether, 17 *emm* types were identified, and a total of 29 subtypes. Four new subtypes were discovered: stG2078.18, stG4222.5, stG6.34, and stG6.35. One isolate remained non-typeable.

**Fig. 1:**
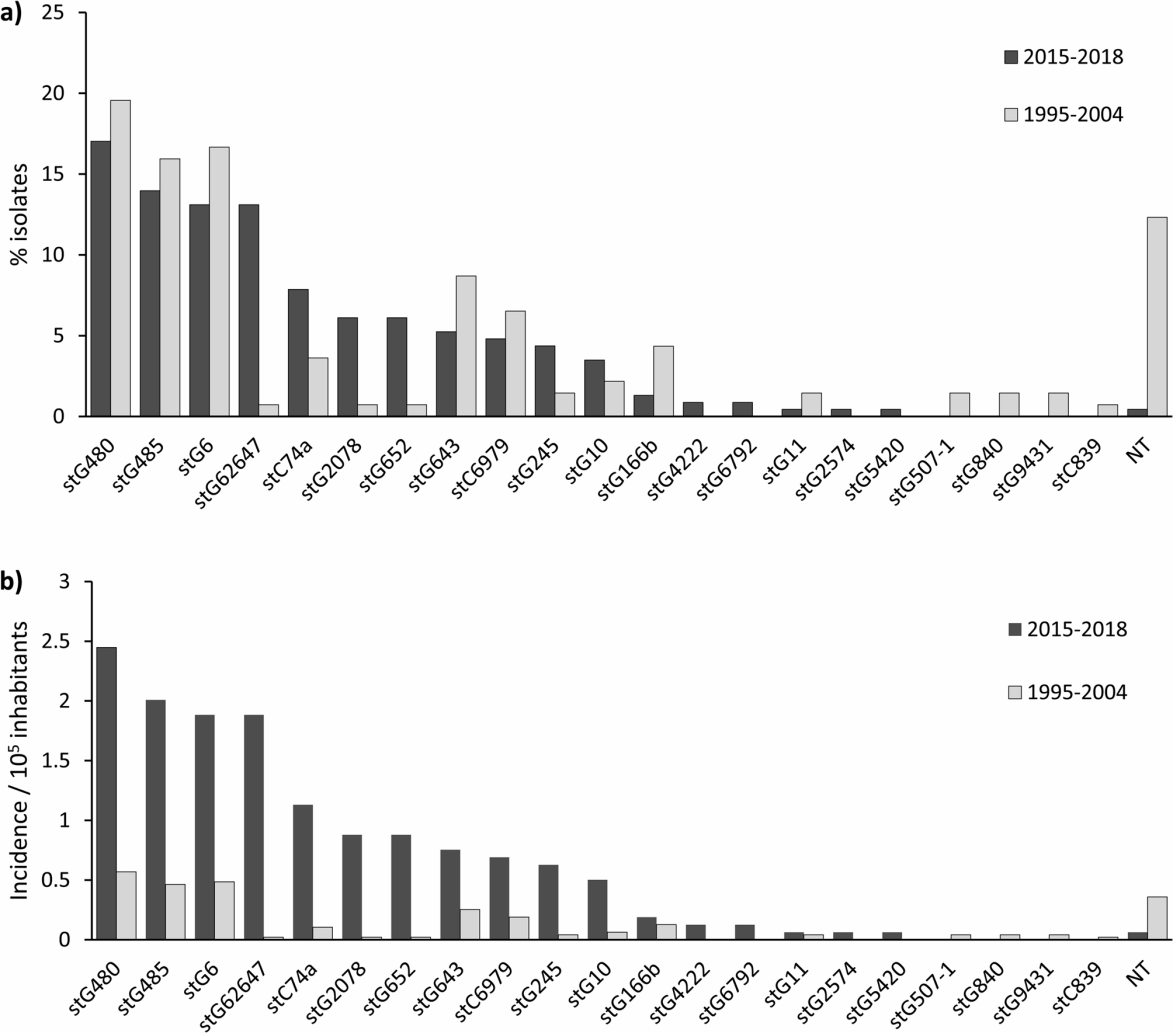
**a** Distribution of 229 *emm* types of *Streptococcus dysgalactiae* subsp. *equismilis* isolates from August 2015 to June 2018, compared with previously published data from the same health district in 1995–2004, Finland. StG480, stG485, and stG6 have remained the three most common *emm* types over the past 20 years, but stG62647 has emerged to share third place with stG6. **b** Mean annual incidence of *emm* types of *Streptococcus dysgalactiae* subsp. *equismilis* isolates from August 2015 to June 2018, compared with a previously published study from the same health district in 1995–2004, Finland. The incidence of all major *emm* types identified in the present study has significantly increased compared to that of 20 years ago. NT, nontypeable

The distribution of *emm* types, compared with findings reported previously from the same health district during 1995–2004, is presented in Fig. 1. The six most prevalent *emm* types identified in the current study were stG480 (39 isolates), stG485 (32 isolates), stG62647 (30 isolates), stG6 (30 isolates), stC74a (18 isolates), and stG2078 (14 isolates), covering 71% of all isolates. Notably, stG480, stG485 and stG6 have remained the three most common *emm* types in the same region over the past 20 years. However, in the present study, stG62647 emerged as the third most common *emm* type, along with stG6, accounting for 13% of the isolates, compared with only one isolate detected over the ten-year period 20 years ago. StG643, stC6979 and stG2078 now constitute smaller proportions compared with the previous data. Conversely, the proportions of stC74a, stG166b, and stG652 have increased. Compared with the previous study, four new types have appeared: stG4222, stG6792, stG2574 and stG5420, and four types have disappeared: stG507-1, stG840, stG9431 and stC839.

Despite changes in distribution, the incidence rates of isolates of all *emm* types identified in this study have significantly increased compared with 20 years ago (Fig. 1b). The incidence of stG62647 isolates has risen dramatically. However, the incidences of the three most common *emm* types in the previous study—stG480, stG485, and stG6— have also all increased, approximately fourfold.

The median age of the patients, in the 217 episodes where medical records were available, was 75 years (range 28 to 95 years), and 60% of the patients were male. The most common clinical manifestations are presented in relation to *emm* types in Table 1. Of all patients, 72% presented with skin and soft-tissue infections (SSTIs). Compared with other *emm* types, bacteremia caused by stG485 more often had an unknown infection focus (*p* = 0.010, OR 3.395 [95% CI 1.375–8.382]), and SSTIs were less common (*p* = 0.046, OR 0.455 [95% CI 0.207–1.000]). Arthritis was more frequently observed in patients with *emm* type stG480 (*p* = 0.033, OR 5.147 [95% CI 1.227–21.589]). Age or sex were not associated with specific *emm* types.

**Table 1:** The most common clinical manifestations and outcome of *Streptococcus dysgalactiae* subsp. *equisimilis* from August 2015 to July 2018, Finland

Site of infection^a^	stG480 *N* = 38				stG485 *N* = 31				stG6 *N* = 29				stG62647 *N* = 28				stC74a *N* = 18					All *N* = 217	
	n	(%)	p-value	OR (95% CI)	n	(%)	p-value	OR (95% CI)	n	(%)	p-value	OR (95% CI)	n	(%)	p-value	OR (95% CI)	n	(%)	p-value		OR (95% CI)	n	(%)
Skin/soft tissue infections	29	(76)	0.593	1.249 (0.552–2.824)	18	(58)	0.046	0.455 (0.207–1.000.207.000)	23	(79)	0.398	1.505 (0.580–3.903)	19	(68)	0.528	0.759 (0.322–1.788)	13	(72)	1.000		0.968 (0.330–2.845)	158	(72)
Cellulitis	27	(71)	0.727	1.147 (0.532–2.473)	17	(55)	0.073	0.497 (0.229–1.078)	22	(76)	0.369	1.510 (0.611–3.727)	17	(61)	0.331	0.667 (0.294–1.515)	10	(56)	0.211		0.540 (0.203–1.434)	149	(69)
Purulent skin infection	7	(18)	0.812	0.897 (0.365–2.202)	4	(13)	0.297	0.558 (0.184–1.691)	9	(31)	0.103	2.038 (0.854–4.865)	3	(11)	0.195	0.447 (0.128–1.556)	5	(28)	0.363		1.630 (0.548–4.848)	43	(20)
Pneumonia	7	(18)	0.672	1.218 (0.488–3.037)	5	(16)	1.000	1.000 (0.356–2.812)	6	(21)	0.429	1.430 (0.536–3.818)	4	(14)	1.000	0.849 (0.275–2.620)	4	(22)	0.502		1.548 (0.478–5.016)	35	(16)
Deep abscess	3	(8)	0.705	1.449 (0.379–5.535)	0		0.223		3	(10)	0.390	2.054 (0.530–7.956)	3	(11)	0.226	2.148 (0.553–8.339)	1	(6)	1.000		0.917 (0.112–7.482)	13	(6)
Bone and joint infection	4	(11)	0.076	3.392 (0.908–12.667)	3	(10)	0.157	2.740 (0.669–11.221)	1	(3)	1.000	0.710 (0.087–5.824)	0		0.367		1	(6)	0.587		1.242 (0.148–10.395)	10	(5)
Arthritis	4	(11)	0.033	5.147 (1.227–21.589)	2	(7)	0.320	2.069 (0.398–10.748)	1	(3)	1.000	0.923 (0.109–7.792)	0		0.600		1	(6)	0.506		1.613 (0.187–13.895)	8	(4)
Bacteremia without defined focus	2	(5)	0.106	0.313 (0.071–1.376)	9	(29)	0.010	3.395 (1.375–8.382)	3	(10)	0.774	0.719 (0.203–2.547)	5	(18)	0.549	1.495 (0.519–4.304)	1	(6)	0.479		0.359 (0.046–2.807)	29	(13)
Fatal outcome	3	(8)	1.000	1.010 (0.276–3.704)	1	(3)	0.478	0.354 (0.045–2.771)	2	(7)	1.000	0.854 (0.185–3.946)	3	(11)	0.466	1.5000 (0.403–5.590)	4	(22)	0.040		4.088 (1.177–14.202)	17	(7)

^a^One patient may have one or more clinical manifestations

The *emm* types accounting for less common clinical manifestations are presented in Table 2. There was notable variation among the *emm* types responsible for these manifestations, with *nine* out of all 16 detected *emm* types also identified in less common manifestations. The four most common *emm* types (stG480, stG485, stG6 and stG62647) were also the most common types in rarer clinical manifestations, collectively accounting for 65% of these episodes, whereas in more common clinical manifestations, the top four *emm* types accounted for 60% of the episodes (*p* = 0.869).

**Table 2:** Distribution of *emm* types among isolates causing rare clinical manifestations of *Streptococcus dysglactiae* subsp. *equisimilis* bacteremia from August 2015 to July 2018, Finland

Site of infection^a^	emm type
Necrotizing fasciitis	1 stG652
Osteomyelitis	2 stG480 1 stG485
Spondylitis	1 stG480 1 stG485
Periprosthetic joint infection	1 stG480 1 stG485 1 stG6 1 stG2078
Empyema	1 stG480 1 stG6
Endocarditis	1 stC74a 1 stG643 1 NT
Aortitis	1 stC74a
Foreign body infection	1 stG480 1 stG62647
Puerperal sepsis	1 stG480 1 stG6 1 stG2078 1 stG6792
Intra-abdominal infection	1 stG480 1 stG485 1 stG62647
Endophthalmitis	1 stG643

^a^One patient may have one or more clinical manifestations

Seventeen patients (8%) died within 30 days of a positive blood culture. As shown in Table 1, the highest case fatality rate was observed in episodes caused by stC74a (22%), which was statistically significantly higher compared with other *emm* types (*p* = 0.040, OR 4.088 [95% CI 1.177–14.202]). Regarding disease severity (admission to an ICU or death within 30 days), no statistically significant differences were observed among the *emm* types. *Emm* types were also studied in relation to various characteristics of severe disease, which included a lowered level of consciousness (or unconsciousness), hypotension, septic shock, disseminated intravascular coagulation, multiorgan failure, and the need for surgical intervention, but no statistically significant differences were observed. *Emm* type StG62647 appeared to be associated with septic shock, though this result did not reach statistical significance (*p* = 0.051). When comparing the eight most common *emm* types (each accounting for over 5% of the isolates) with the rarer *emm* types, no statistically significant differences were found in terms of case fatality rate (9% vs. 3%, *p* = 0.317) or disease severity (12% vs. 3%, *p* = 0.136). Similar results were found in comparison of the top four *emm* types (each accounting for over 10% of the isolates) vs. others.

All *emm* types were susceptible to penicillin and cephalosporins. Clindamycin resistance was observed in 2% of strains, and one strain (0.4%) was intermediately sensitive. All strains resistant to clindamycin were also resistant to erythromycin. The rate of erythromycin resistance was 10%, and 2% were intermediately sensitive. The highest rates of resistance to both clindamycin and erythromycin were found in connection with stG480 (*n* = 38, 16% each) and stG245 (*n* = 8, 13% and 25% respectively). Among the ten most common *emm* types, clindamycin resistance was observed in stG485 (*n* = 31, 3%) and stG652 (*n* = 12, 8%). Erythromycin resistance was present in all ten most common *emm* types, but not in stG62647 (*n* = 28), stG10 (*n* = 8), and stG166b (*n* = 3). Notably, there was no significant resistance in the emerging *emm* type stG62647; one strain was intermediately sensitive to erythromycin and all strains were susceptible to clindamycin.

## Discussion

In this population-based study, the three most common *emm* types remained stG480, stG485 and stG6, consistent with findings from the same geographic region 20 years ago. StG485 and stG6 are prevalent in several countries globally [15, 22, 23, 27–29]. StG480 is frequent in Norway, Sweden and Austria, as in Finland, but is rarer in other countries [15, 16, 27]. In the USA, stG6 has been the most common *emm* type, and stG485 and stG480 ranked among the top eight [30]. Interestingly, none of these three *emm* types were among the most common in Canada [21]. 

The most notable change in *emm*-type distribution in our study was the emergence of *emm* type stG62647, which is now, along with stG6, the third most common type. This trend has been observed in several other countries. In Norway, stG62647 was rare prior to 2013, but between 2013 and 2015, it became the most common *emm* type, representing 20% of the isolates [17]. Increasing proportions of stG62647 have also been reported in Sweden, Denmark, Germany, Spain, and Canada [16, 18–21]. Conversely, in Japan, the lineage stG6792, which was rare before 2003, has since become the most common *emm* type [5]. In. a Norwegian study concerning the geographic distribution of SDSE sequence types, it was found that the SDSE population in Norway and Canada shared similar sequence types, while the SDSE population in Japan was more phylogenetically divergent [31]. 

There are only a few previous studies concerning the associations between *emm* types and disease severity. StG62647 has been associated with severe clinical outcomes, including necrotizing soft-tissue infections, endocarditis, streptococcal toxic shock syndrome, and death, when compared with other *emm* types [17]. In a mouse model of necrotizing myositis, stG62647 was shown to be significantly more virulent than *emm* type stC74a [32]. However, in our study, stG62647 was not significantly associated with severe disease. While the incidence of septic shock was higher in connection with stG62647 compared with other emm types, this difference did not reach statistical significance. In contrast, in our study, stC74a was significantly associated with higher mortality rates. Some investigators have found stC74a to be more prevalent among invasive isolates compared with non-invasive ones [19, 27]. In a Swedish study of SDSE endocarditis, stC74a and stG62647 were the most common *emm* types, whereas in all invasive isolates in Sweden, stC74a ranked only as the seventh most common [16, 33]. In Japan, *emm* type stG6792 has been associated with poor clinical outcomes [22]. In another study, *emm* types stG2078 and stG10 were associated with invasive disease, while stG6792 and stG166b were linked to non-invasive disease [34]. However, many studies concerning *emm* types and disease severity in SDSE bacteremia are limited by small sample sizes or insufficient availability of clinical data. More research is needed to better understand the associations between *emm* types and disease severity in SDSE bacteremia.

In a previous study conducted within the same health district as the current study, rarer *emm* types were associated with severe disease, compared with the five most common ones [14]. Similar findings were reported in Norway, where mortality rates were higher in connection with less common *emm* types [15]. However, in our current study we did not confirm these results. Instead, we observed that the most prevalent *emm* types appeared to cause more severe disease and higher case fatality rates, although the differences were not statistically significant. Additionally, our results suggested that even in cases of rarer clinical manifestations, the majority were still caused by the most common *emm* types.

Both previous studies in Finland and Norway in which rare *emm* types were associated with severe disease were conducted when the incidence of SDSE bacteremia was much lower, and before the emergence of lineage stG62647. Since then, the incidence rates of both previously common *emm* types and that of stG62647 have risen significantly. It is possible that these common *emm* types, along with stG62647, have acquired selective advantages, leading to their dramatic increase in incidence and shifting the spectrum of *emm* types causing severe disease toward the more common ones.

In the same geographic area as the present study, the incidence of SDSE bacteremia increased from 2.05 to 4.75 episodes per 100,000 inhabitants between 1995 and 2004 [35]. As reported in our previous article, the mean annual incidence during the current study period reached 16.9 per 100,000 inhabitants [10]. The substantial rise in the incidence of SDSE bacteremia in Finland appears to be due to the increased prevalence of common *emm* types, along with the emergence of stG62647. In addition to potential microbial adaptations, changes in host susceptibility are likely to have contributed to these trends. SDSE primarily causes bacteremia in elderly individuals with multiple comorbidities. In our previous study we identified obesity, diabetes, and coronary artery disease as significant risk factors of SDSE bacteremia. Over the past two decades in Finland, there has been a notable increase in the prevalence of obesity and diabetes, along with a rise in the proportion of individuals aged above 65 years [36–38]. 

As well as disease severity, previous research on the associations between SDSE *emm* types and clinical manifestations is limited, with only a few descriptive studies available. In one study no association was found between *emm* types and clinical outcomes [23]. Regarding *S. pyogenes*, however, certain *emm* types have shown associations with specific conditions: *emm1* and *emm3* have been linked to severe diseases such as necrotizing fasciitis and streptococcal toxic shock syndrome, and *emm28* to puerperal sepsis [39]. In our study, stG480 was associated with arthritis, while stG485 more often had an unknown infection focus and it was less frequently associated with skin and soft-tissue infections. No other statistically significant associations between *emm* types and clinical manifestations were observed. Further studies are needed to determine whether these findings can be confirmed in other populations.

In our study, we found decreased susceptibility (intermediate susceptibility or resistance) to erythromycin in 12% of strains, and to clindamycin in 2%. A comparative analysis covering 1995 to 2004 in the Pirkanmaa Health District revealed decreased susceptibility to erythromycin in 10% of Group G Streptococcus isolates and a 1% decrease as regards clindamycin [9]. However, in other countries, rising trends in erythromycin and clindamycin resistance in SDSE have been reported. In Norway, resistance to both erythromycin and clindamycin, which was previously absent before 2009, increased to 12% during 2016–2018 [40]. Similarly, in Japan, macrolide resistance grew from 10.3% in 2003–2005 to 18.5% in 2010–2013[5, 41].

Previous investigators have reported significant diversity in *emm* types with respect to antimicrobial susceptibility. In Japan, *emm* types stG245 and stG10 have been associated with macrolide resistance [5]. In our study, the highest rates of erythromycin and clindamycin resistance were observed in lineage stG245, but no resistance was detected in lineage stG10.

In South Korea, *emm* type stG254 has been linked to resistance to both macrolides and erythromycin [42]. 

Our study has several strengths. The study population is relatively large, and it combines *emm* types with comprehensive clinical data. As a population-based study, it provides reliable distributions of both *emm* types and clinical manifestations of SDSE bacteremia. The *emm* typing was thorough, with only one strain remaining non-typeable. Additionally, *emm* typing had been performed in the same geographic region two decades earlier, allowing for a detailed understanding of changes in *emm* type distribution.

There are also some limitations. To better assess the associations between *emm* types and clinical manifestations, an even larger study population would be beneficial. In future, more studies of associations between *emm* types, clinical manifestations and disease severity are needed.

## Conclusion

In this population-based study, the most common *emm* types causing SDSE bacteremia—stG485, stG480, and stG6—remained consistent with findings from the same geographic region 20 years ago. The most notable change in *emm*-type distribution was the emergence of stG62647 as the third most common *emm* type, along with stG6. However, the dramatic rise in the incidence of SDSE bacteremia over the past few decades is not solely due to the emergence of stG62647—the incidence of all major *emm* types has increased significantly, contributing to the overall surge in SDSE bacteremia. In our study, *emm* type stC74a was found to be significantly associated with mortality. More studies concerning the associations between *emm* types, disease severity and clinical manifestations are needed.

## Data Availability

The datasets generated and/or analyzed during this study are not publicly available due to the protection of participant confidentiality. For inquiries regarding the datasets or requests for additional analyses, please contact the corresponding author.
